# Levofloxacin-induced acute psychosis

**DOI:** 10.4103/0019-5545.39762

**Published:** 2008

**Authors:** Nagaraja Moorthy, N. Raghavendra, P. N. Venkatarathnamma

**Affiliations:** Department of General Medicine, Sri Devaraj Urs Medical College and RLJ Hospital and Research Centre, Tamaka, Kolar, Karnataka, India

**Keywords:** Acute psychosis, fluoroquinolones, levofloxacin

## Abstract

A wide range of drugs can cause mental status changes. Fluoroquinolones are one among them and are underrecognised.The CNS side effects of levofloxacin like headache, dizziness, restlessness, tremor, insomnia, hallucinations, convulsions, anxiety and depression are well documented. We report a rare case of middle aged diabetic male admitted to hospital with multiple infections who developed acute psychosis following levofloxacin administration.

## INTRODUCTION

Fluoroquinolones are an under-recognized cause of changes in mental status. Delirium and hallucinations associated with fluoroquinolones have been reported, particularly with ciprofloxacin.[1] The proposed mechanism involved in the development of such side effects seems to be related to the quinolones’ ability to inhibit the binding of y-aminobutyric acid (GABA) to the GABA receptors, leading to central nervous system (CNS) excitation.[2] We report here a rare case of levofloxacin-induced acute psychosis encountered during treatment of a diabetic adult with multiple infections.

## CASE HISTORY

A 50-year-old man, nonalcoholic, with uncontrolled diabetes mellitus and hypertension was admitted to the hospital with history of high-grade fever and cough with scanty expectoration, of 2 days’ duration; and burning micturition and ulcer over left foot. Clinically the patient was febrile and diagnosed to have community-acquired left lower lobe pneumonia with urinary tract infection and cellulites of left foot. Investigations revealed Hb was 10.4 g/dl, total leukocyte count was 9,500 cells/mm^3^ with neutrophilia, E.S.R. was 60 mm at one hour, random blood sugar was 250 mg/dl, blood urea was 25 mg/dl, serum creatinine was 1.3 mg/dl with normal creatinine clearance, and serum electrolytes were within normal limits. Peripheral smear for malarial parasite was negative. Urine microscopy showed 15-18 pus cells/high power field. However, urine culture was sterile and urine ketone bodies were negative. Blood and sputum culture did not grow any organisms. Final diagnosis of type 2 diabetes mellitus with hypertension with community-acquired pneumonia and urinary tract infection and cellulites of left foot with ulcer was made. In view of multiple infections, intravenous amoxicillin (1 g) and clavulanic acid (200 mg) every 8^th^ hour were started and continued for 10 days. His general condition improved, and repeat chest x-ray showed resolution of pneumonia with better lung aeration. Cellulitis and urinary tract infection also showed improvement, and blood sugar and hypertension were under control. After 10 days, oral levofloxacin (500 mg/day) was started as a sequential therapy in view of persisting foot ulcer. On the third day of therapy, he became restless and speech became irrelevant and incoherent. Later he became abusive, violent and experienced visual hallucinations of people in his hospital room. Gradually his confusion worsened and he became more violent in nature. He slept very little. Psychiatric evaluation was suggestive of acute psychosis. The diagnosis of acute psychosis cannot be attributed to the clinical diagnosis as the patient had good improvement following 10 days of intravenous amoxicillin and clavulanic acid therapy. Other conditions like hypoglycemia, dyselectrolytemia, diabetic ketoacidosis, and meningitis were ruled out. Other drugs the patient was receiving were insulin, enalapril, atorvastatin, which are not known to result in such psychosis. So the likely possibility of levofloxacin-induced acute psychosis was considered and levofloxacin was stopped. Within 48 h of stopping levofloxacin, repeat psychiatric evaluation revealed him to be alert and oriented with no further hallucinations. His speech was normal in flow and content, and his concentration and recall were intact. He did not require any antipsychotic medications.

## DISCUSSION

Levofloxacin is a third-generation fluorinated quinolone antibiotic which is an optically active I-isomer of the racemate ofloxacin, having a broad spectrum of antibacterial activity against both gram-positive and gram-negative bacteria. It exerts its antibacterial activity via antagonism of interaction between bacterial DNA gyrace and DNA.[3] The tolerance profile of levofloxacin can be considered to be good; rather better than most, if not all, of the other fluoroquinolones available. The major reported adverse effects of fluoroquinolones are gastrointestinal (3-17%) and central nervous system-related (0.9-11%) disturbances. The CNS-related side effects of levofloxacin are headache, dizziness, restlessness, tremor, insomnia, hallucinations, convulsions, anxiety, and depression.[4,5] The development of these effects seems to be related to the degree to which the fluoroquinolones bind to GABA receptor and their differing potential to act as GABA antagonist and bind to the N-methyl-D-aspartate receptor. According to the European dossier data, from 5,388 patients treated with levofloxacin, 12% of patients developed an adverse effect, possibly related to the drug; but only 1% of these were classified as serious. Treatment with levofloxacin was discontinued in only 4%. Psychosis occurred in only 1/6 million prescriptions.[6] Neurotoxicity of fluoroquinolones is an important problem that has to be considered with this group of antibiotics.

## CONCLUSION

Fluoroquinolones are widely used in the treatment of various infections; hence it is important to be aware of adverse effects of these drugs, which should be used for treatment only under close supervision. This case report suggests that caution should be exercised when administering newer fluoroquinolones like levofloxacin.
